# A case report of gastric serrated adenoma arising in a patient with bile reflux gastritis and related literature review

**DOI:** 10.3389/fonc.2025.1670442

**Published:** 2025-10-06

**Authors:** Chang Cai, Liping Yan, Kuang-I Fu, Bin Ye, Yixiu Shao, Han Wang, Qin Xu, Lixia Fu

**Affiliations:** ^1^ Department of Gastroenterology, The Fifth Affiliated Hospital of Wenzhou Medical University and Lishui Central Hospital, Lishui, China; ^2^ Department of Pathology, The Fifth Affiliated Hospital of Wenzhou Medical University and Lishui Central Hospital, Lishui, China; ^3^ Department of Endoscopy, Kanma Memorial Hospital, Tochigi, Japan

**Keywords:** gastric, serrated adenoma, gastric serrated adenoma, serrated lesions, bile reflux, gastric cancer

## Abstract

Serrated adenoma is often found in the colorectum, but seldom found in stomach. We reported a case of a 70-year-old male with gastroscopy showed an elevated lesion with central depression on the gastric body, with clear margins. Background consistent with bile reflux gastritis. Endoscopic submucosal dissection (ESD) was performed. Histopathology confirmed a gastric serrated adenoma. This study reports the first documented case of gastric serrated adenoma arising in a background of bile reflux gastritis, suggesting bile reflux may be a potential risk factor for gastric serrated adenoma development.

## Introduction

Serrated adenoma predominantly occurs in the colorectum. Endoscopically, it often manifests as a flattened, misty appearance. Histopathologically, it is characterized by dilatation of the base of the crypts which often grow parallel to the muscularis mucosa forming L shaped or inverted T-shaped crypts (1), representing an important pathway to colorectal cancer. Gastric serrated adenoma was first reported by Rubio in 2001 (2) and is exceptionally rare, with an estimated incidence of 0.017% (3). However, gastric serrated adenoma exhibits high aggressiveness, with 74.3% showing submucosal invasion at diagnosis (4). The background mucosa associated with its development remains unclear. This report describes a case of gastric serrated adenoma confined to the mucosa arising from bile reflux gastritis.

## Case report

**Figure 1:**
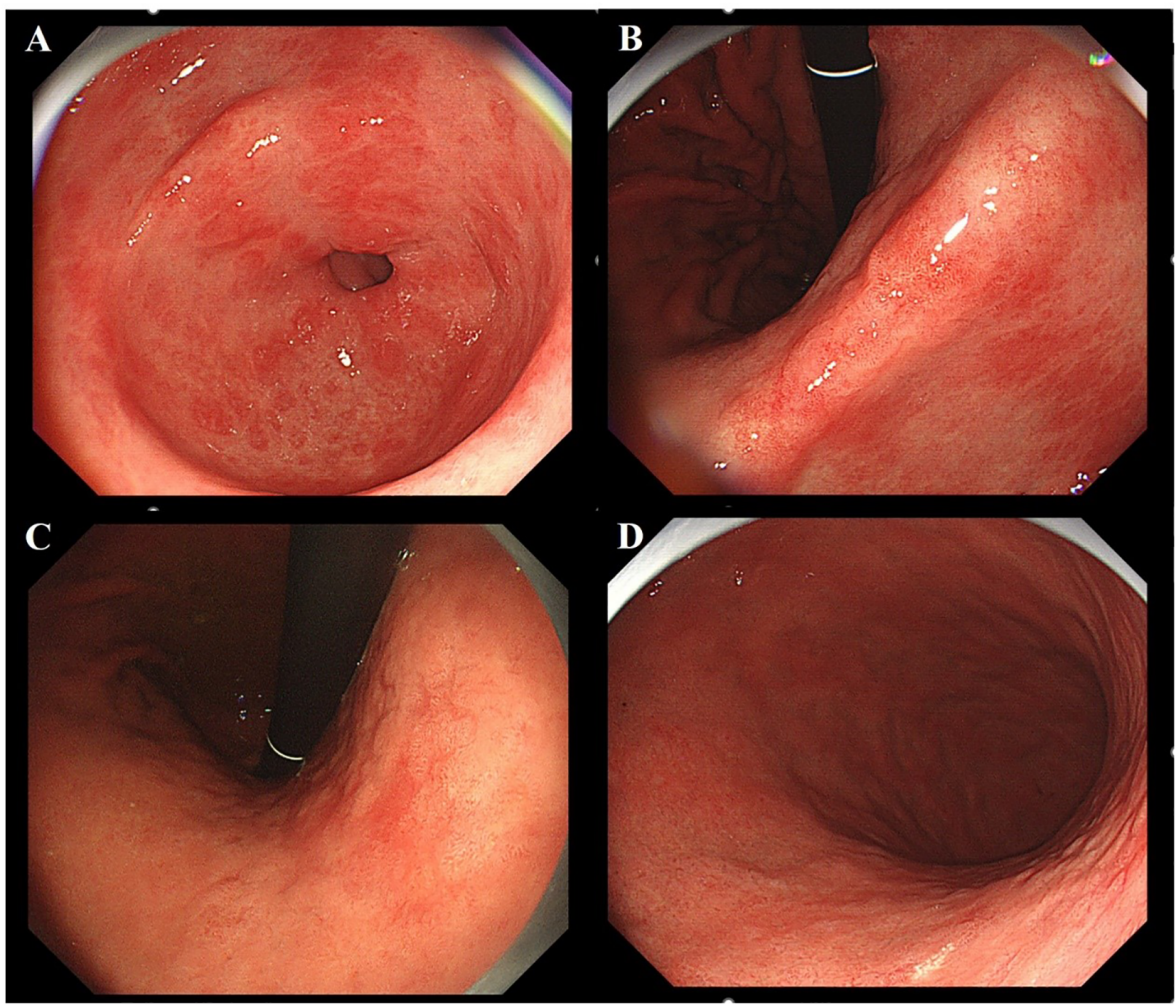
Analysis of lesion background mucosa. **(A)** Multiple linear erosions are seen around the pylorus. **(B, C)** Map-like redness is observed in the gastric body and gastric angle. **(D)** No atrophic demarcation line was observed at the antrum-body junction of the stomach.

**Figure 2:**
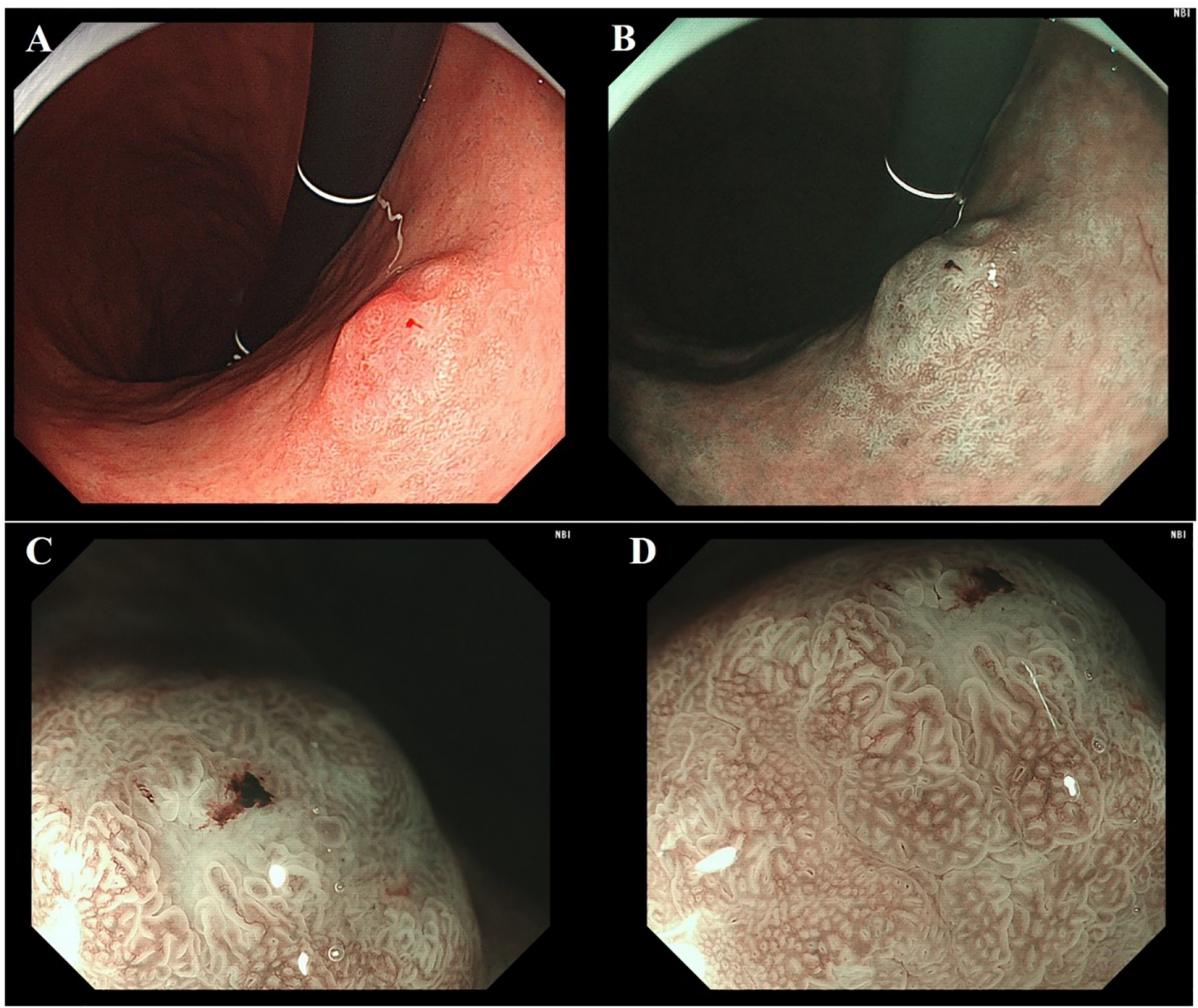
Endoscopic features of gastric traditional serrated adenoma. **(A)** Under white light, the lesion appears pink with clear demarcation line. **(B)** The lesion under NBI appears as grayish-white changes. **(C, D)** Under NBI+ME, the glandular ducts appear elongated with serrated changes and internal spiral vessels.

A 70-year-old male with a history of diabetes mellitus and hypertension was admitted following gastroscopy for acid reflux and belching. The previous C-14 urea breath test was negative, and there was no history of eradication of Helicobacter pylori (H. pylori). Gastroscopy revealed multiple patchy erosions around the pylorus (**Figure 1A**) and map-like redness in the gastric angle and body (**Figure 1B, C**). No atrophic border was observed (**Figure 1D**). A 1.0 cm type IIa+IIc lesion was identified on the posterior wall of the lower gastric body. Under white light, the lesion appeared pale with demarcation line and a slightly depressed central area (**Figure 2A**); it exhibited deformation upon air suction. Narrow-band imaging (NBI) showed a grayish-white surface without significant brownish areas (**Figure 2B**). Narrow-Band Imaging-Magnifying Endoscopy (NBI+ME) revealed densely packed, papillary-elongated glands, dilation of the intervening part, peripheral serration, and internal spiral vessels (**Figures 2C, D**). The lesion was suspected of a mucosal neoplasm, and ESD was performed.

**Figure 3:**
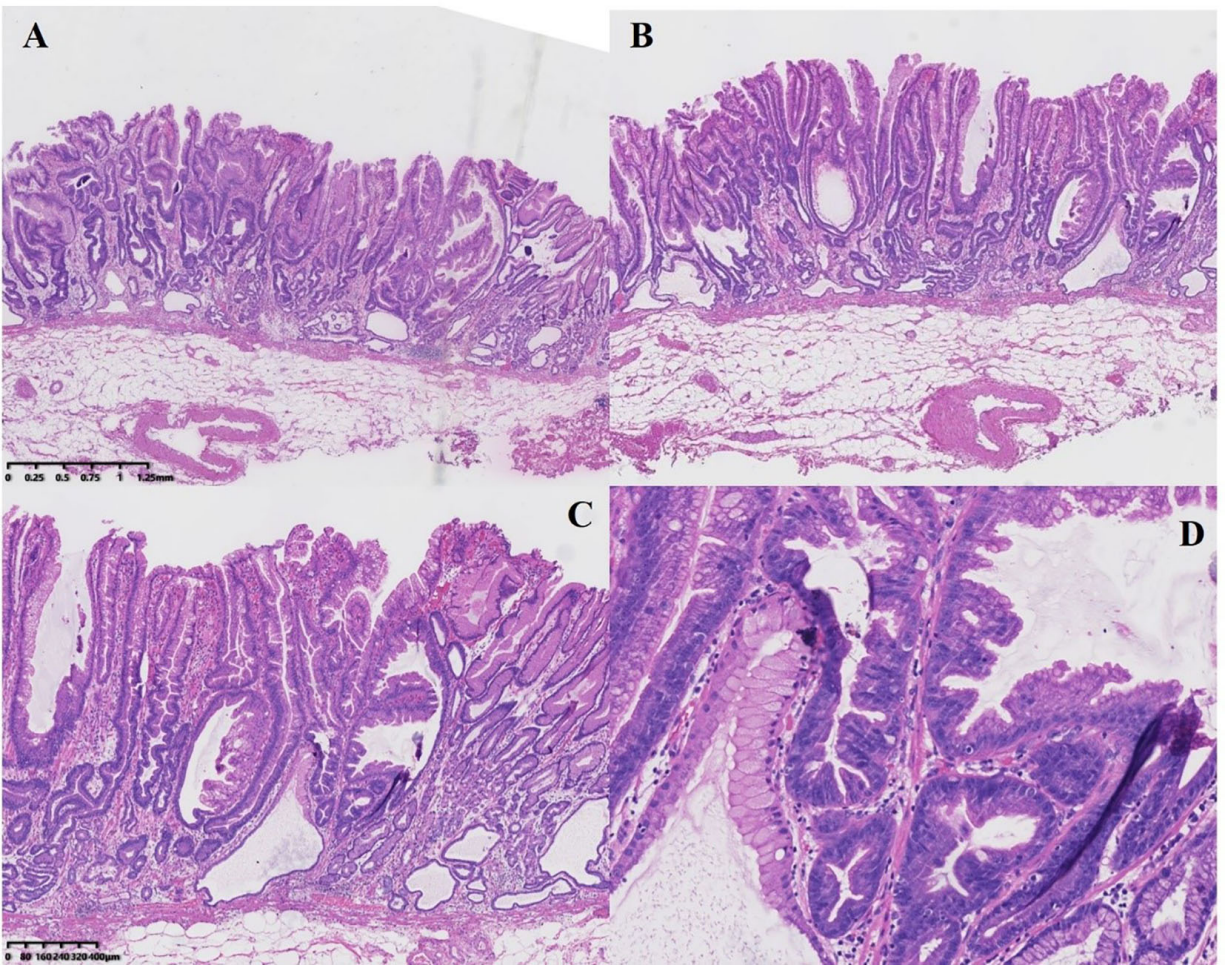
Pathological features of the serrated adenoma by hematoxylin & eosin (HE). **(A, B)** The cytoplasm of the lesion area shows eosinophilic changes, with glandular ducts presenting serrated and slit-like alterations. The lesion is confined to the mucosal layer. **(C)** Glands showed “inverted T-shaped” configurations. **(D)** Centrally placed pencillate nuclei.

Histologically, the background mucosa exhibited changes consistent with bile reflux gastritis. The tumor was sharply demarcated from the surrounding mucosa. Tumor cells were tall columnar, resembling gastric foveolar epithelium, arranged in serrated alteration predominantly at the base. Some glands showed “inverted T-shaped” configurations. Tumor cells had abundant cytoplasm and were partially eosinophilic, with centrally placed pencillate nuclei. The lesion was confined to the mucosa without lymphovascular invasion or perineural involvement (**Figures 3A–D**).

**Figure 4:**
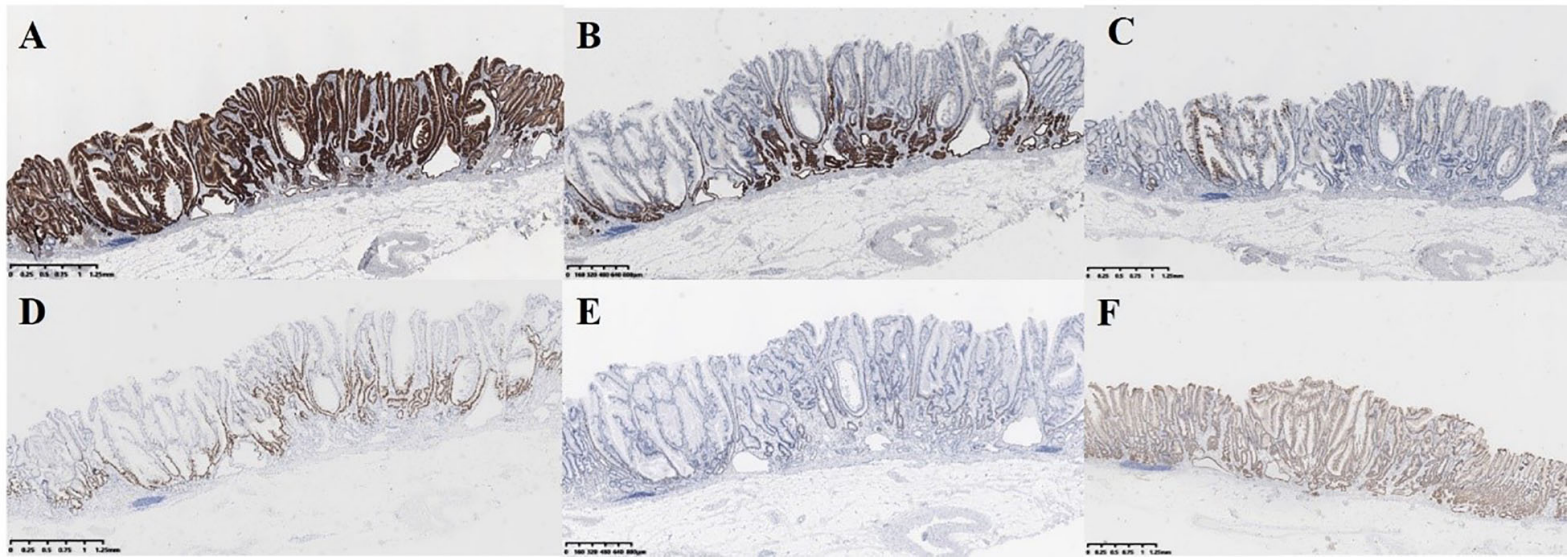
Pathological features of the serrated adenoma by immunohistochemical staining (IHC). **(A)** MUC5AC diffuse positivity throughout the tumor. **(B)** MUC6 was positive in basal glands. **(C)** MUC2 showed focal positivity. **(D, E)** The Ki-67 and p53 were positive in basal glands. **(F)** β-Catenin showed membranous positivity.

Immunohistochemical staining showed MUC5AC diffuse positivity throughout the tumor (**Figure 4A**) MUC6 was positive in basal glands. (**Figure 4B**) MUC2 showed focal positivity. (**Figure 4C**) This mucin phenotype (gastric-type predominant, mixed gastrointestinal) supported serrated adenoma. The ki-67 and p53 were positive in basal glands. (**Figures 4D, E**) β-Catenin showed membranous positivity (**Figure 4F**). Genetic testing shows that No *BRAF V600E* or KRAS mutations were detected. The lesion was histopathologically confirmed as serrated adenoma in the lower gastric body, posterior wall, size 0.6 cm x 1.0 cm, exhibiting partial low-grade dysplasia and focal high-grade dysplasia (WHO criteria)/Gastric-type well-differentiated adenocarcinoma (papillary growth, foveolar-type predominant, Japanese criteria), the surgical margins was negative. Background was consistent with bile reflux gastritis.

## Discussion

Serrated lesions are relatively common in the colon and represent a significant pathway to colorectal cancer, accounting for approximately 30% of cases (5). In contrast, gastric serrated lesions are rare. A retrospective study of 98,746 gastroscopies identified only 21 upper gastrointestinal serrated lesions, 17 of which were gastric, indicating a very low incidence (3). Despite this rarity, gastric serrated lesions is highly aggressive, with 74.3% showing submucosal invasion at diagnosis (4). The present case was detected early and confined to the mucosa. Notably, among 6 gastric serrated lesions larger than 4 cm reported by Kwon et al. (6), 4 invaded at least the submucosa, suggesting a potential positive correlation between lesion size and invasion depth. Intriguingly, the risk of developing colon cancer in patients with serrated lesions of the colon is approximately 1.52-4.2 times higher than that of normal individuals (7–9), significantly lower than the aggressiveness observed in gastric serrated lesions. This stark difference remains unexplained but may relate to the low detection rate of gastric serrated lesions, insufficient recognition by endoscopists and pathologists, and potential misdiagnosis as villous adenomas (10).

Crypt dilation, irregularly branching crypts, and horizontally arranged basal crypts (inverted T- and/or L-shaped crypts are hallmark features of serrated adenoma (11). However, not all serrated adenoma exhibit all three features simultaneously. Current diagnostic criteria require at least two of these features. The lesion showed inverted T-shaped” configurations and crypt dilation. aligning with diagnostic criteria.

Hasuo et al. (12) reported a case of serrated lesions detected in the context of *Helicobacter pylori* (HP) eradication, while García et al. (13) described a lesion occurring in an HP-negative background. Köklü et al. (14) identified serrated adenoma in a patient with active HP infection. Other researchers did not specify HP status. This variability leaves the association between HP infection and gastric serrated adenoma unresolved. Furthermore, studies report an inverse correlation between HP infection and the occurrence of colorectal serrated lesions (15). Unlike most gastric neoplasms, we hypothesize that gastric serrated adenoma development may be independent of HP infection. Although the background mucosa exhibited a geographical pattern in the present case, no distinct atrophic border was observed. The patient’s C-14 urea breath test was negative, confirming no history of HP infection or eradication therapy. Therefore, we consider the lesion’s development unrelated to HP infection.

Prominent linear erosions near the pylorus suggested bile reflux. Post-ESD pathological examination of the surrounding mucosa revealed mild inflammation, minimal atrophy, spiral hyperplasia of the foveolar epithelium, and longitudinal growth of the muscularis mucosae – characteristic morphological features of bile reflux gastritis. Thus, this gastric serrated adenoma arose against a background of bile reflux gastritis.

Bile reflux is a known causative factor for intestinal metaplasia and increases the risk of gastric cancer (16, 17). Elevated bile acid concentrations correlate positively with gastric cancer risk, likely mediated through mechanisms such as the activation of PKC and COX-2 by bile acids like chenodeoxycholic acid and the significant role of the bile acid receptor TGR5 in promoting epithelial-mesenchymal transition (EMT) in gastric cancer (GC) cells. Previous studies on gastric cancer arising in the context of bile reflux have not further subtyped the pathology, nor have they reported associated serrated adenoma. The relationship between serrated adenoma and bile reflux remains undefined and warrants further investigation.

Although long-term prognosis data for gastric serrated adenoma are lacking, the US Multi-Society Task Force (18) and the European Society of Gastrointestinal Endoscopy (19) recommend resection for colorectal sessile serrated lesions followed by colonoscopy surveillance every 3 years. Furthermore, this lesion met the criteria for endoscopic submucosal dissection (ESD) (20), therefore, ESD was performed.

In conclusion, this represents the first reported case of gastric serrated adenoma confined to the mucosa arising in a background of bile reflux gastritis. It offers valuable insights for future research into this rare entity.

## Data Availability

The raw data supporting the conclusions of this article will be made available by the authors, without undue reservation.
